# Meteorological Register for April, 1817

**Published:** 1817-06

**Authors:** John Bailey

**Affiliations:** Surgeon, &c.


### METEOROLOGICAL REGISTER FOR APRIL, 1817,

*Kept at Harwich, in Essex,*

By JOHN BAILEY, Surgeon, &c.

| Moon's Age. | | Atmospherical Preſſure. Morn. | Ev. | Temp. | Wind. | Remarks. | General Statement of Diſeaſes. |
|---|---|---|---|---|---|---|---|
| ○ | 1 | 30 4½ | 30 4½ | 47 | S | Foggy | |
| | 2 | 3 | 3 | 50 | E | Fine | |
| | 3 | 2 | 2 | 55 | SE | ditto | |
| | 4 | 3 | 3 | 45 | E | ditto | |
| | 5 | | | 43 | E | ditto | |
| | 6 | | | 43 | NE | Dull & cloudy | |
| | 7 | 3½ | | 49 | E | | |
| ☾ | 8 | 1½ | 29 8½ | 52 | SW | Snow. G. of wind | |
| | 9 | 29 8½ | | 50 | NE | | |
| | 10 | 9 | 30 ½ | 37 | NE | Rain | |
| | 11 | 30 1½ | 1½ | 33 | NE | Dull & cloudy | |
| | 12 | 29·9½ | 29 9½ | 50 | N | | |
| | 13 | 30 | 30 | 50 | N | | |
| | 14 | 29 9½ | 29 9½ | 50 | N | Rain. Gale of wind | |
| | 15 | 8½ | 7½ | 59 | S | Snow, ditto | |
| ● | 16 | 6 | 9 | 50 | N | | |
| | 17 | 30 | 30 1½ | 44 | N | | |
| | 18 | 2 | 3 | 49 | SE | | |
| | 19 | 3 | | 57 | SE | | |
| | 20 | | | 51 | NE | | |
| | 21 | | 2 | 55 | E | | |
| | 22 | 2 | 2 | 51 | E | | |
| | 23 | 1½ | 1½ | 47 | NE | | |
| ☽ | 24 | | | 50 | NE | Rain | |
| | 25 | 1 | 1 | 45 | NE | ditto | |
| | 26 | 29 9½ | 29 8 | 46 | E | Hail | |
| | 27 | 9 | 9 | 47 | E | Dull & cloudy | |
| | 28 | 30 ½ | 30 | 51 | SW | ditto | |
| | 29 | 29 9 | 29 7 | 51 | NW | ditto | |
| | 30 | 5½ | 5½ | 50 | NW | Rain | |

The observations which accompanied the last Register may be fairly applied to this. An isolated case of Rubeola sine Catarrho, occurred in a child.—An unusual exemption from wet may be considered as the cause of the present healthfulness.

---

END OF VOL. III.

